# Text Authorship Identified Using the Dynamics of Word Co-Occurrence Networks

**DOI:** 10.1371/journal.pone.0170527

**Published:** 2017-01-26

**Authors:** Camilo Akimushkin, Diego Raphael Amancio, Osvaldo Novais Oliveira

**Affiliations:** 1 São Carlos Institute of Physics, University of São Paulo, São Carlos, São Paulo, Brazil; 2 Institute of Mathematics and Computer Science, University of São Paulo, São Carlos, São Paulo, Brazil; Tianjin University, CHINA

## Abstract

Automatic identification of authorship in disputed documents has benefited from complex network theory as this approach does not require human expertise or detailed semantic knowledge. Networks modeling entire books can be used to discriminate texts from different sources and understand network growth mechanisms, but only a few studies have probed the suitability of networks in modeling small chunks of text to grasp stylistic features. In this study, we introduce a methodology based on the dynamics of word co-occurrence networks representing written texts to classify a corpus of 80 texts by 8 authors. The texts were divided into sections with equal number of linguistic tokens, from which time series were created for 12 topological metrics. Since 73% of all series were stationary (ARIMA(p, 0, q)) and the remaining were integrable of first order (ARIMA(p, 1, q)), probability distributions could be obtained for the global network metrics. The metrics exhibit bell-shaped non-Gaussian distributions, and therefore distribution moments were used as learning attributes. With an optimized supervised learning procedure based on a nonlinear transformation performed by Isomap, 71 out of 80 texts were correctly classified using the K-nearest neighbors algorithm, i.e. a remarkable 88.75% author matching success rate was achieved. Hence, purely dynamic fluctuations in network metrics can characterize authorship, thus paving the way for a robust description of large texts in terms of small evolving networks.

## 1 Introduction

Statistical methods have long been applied to analyze many complex systems [1–5], including written texts and language patterns [6], which now include network representations of text to investigate linguistic phenomena [7–14]. Networks generated from text share several features with other complex systems, e.g. information and transportation networks [15, 16], biological systems [17, 18], and social interactions [19]. Examples of language-related networks include phonological networks with modular or cut-off power-law behaviors [20–23], semantic similarity networks with small-world and scale-free properties [24], syntactic dependency networks with hierarchical and small-world organization [25, 26] and collocation networks, which also display small-world and scale-free properties [8]. The ubiquity of specific patterns in language networks is believed to account for an easy navigation and acquisition in semantic and syntactic networks [27]. Of particular relevance to this study, word co-occurrence networks are a special case of collocation networks where two words (nodes) are linked if they appear close to each other in a text. Co-occurrence networks are convenient because they do not require prior linguistic knowledge, apart from that needed to filter relevant information. Since most of the syntactic relations occur between adjacent words, co-occurrence networks can be seen as simplified versions of syntactic networks [26]. Several patterns have been identified in co-occurrence networks formed from large corpora, such as the power-law regimes for degrees distribution [7] and core-periphery structure [28] resulting from the complex organization of the lexicon. The overall structure and dynamics of networks representing texts have been modeled to describe their mechanism of growth and attachment [29, 30], while nuances in the topology of real networks were exploited in practical problems, including natural language processing [31–34]. In this study, we use the co-occurrence representation to probe how the variation of network topology along a text is able to identify author’s style.

Writing style is more subjective than other text characteristics (e.g. topic), making authorship recognition one of the most challenging text mining tasks [35, 36]. It is crucial for practical applications such as text classification [34], copyright resolution [37], identification of terrorist messages [38] and of plagiarism [35]. Early studies using stylometry were conducted by Mosteller and Wallace to identify authorship of the Federalist Papers [39]. A myriad of methods to tackle the problem have been developed since then, typically using statistical properties of words (e.g. mean length, frequency, burstiness and vocabulary richness) and characters (e.g. character counts and long-range correlations), in addition to syntactic and semantic information, and text format [35]. Methods from statistical physics have also been used for authorship recognition [40, 41], which in recent years included text modeling with co-occurrence networks [42–47]. The adequacy of co-occurrence networks for the task was confirmed for the first time with the correlation between network topology and authors’ styles [34]. Despite this relative success, some issues concerning the applicability of network methods remain unsolved.

A major issue in network representation is that regular patterns among concepts only emerge when large pieces of text are available. Furthermore, rigorous network-based similarity estimators usually assume that the networks comprise the same number of nodes and edges, since most measurements are affected by the network size [48]. Unfortunately, such strong assumption often does not hold for written texts ranging from small tales to long novels, which may hinder the applicability of models to real situations. Additionally, since characterization of networks hinges on a precise statistical analysis, a large number of networks with similar characteristics need to be analyzed. As we shall show, the method presented here obviates these issues with a simple approach based on network dynamics.

## 2 Methods

The modelling of real systems using a time series approach plays a prominent role in many applications [49–53]. Written texts were represented as sets of co-occurrence networks, from which network dynamics measurements were obtained. These measurements were used as attributes in pattern recognition methods in order to identify the author of a given text. The construction and analysis of the measurements are described in detail in the following subsections.

### Modeling texts as co-occurrence networks

The texts used for classification come from a collection of novels and tales in English whose details are provided in the Supporting Information (S1 File). The collection comprising 8 authors with 10 texts per author was selected to simulate a real case where the text lengths are varied in a range from 2,853 to 267,012 tokens with an average of 53,532 tokens.

**Fig 1:**
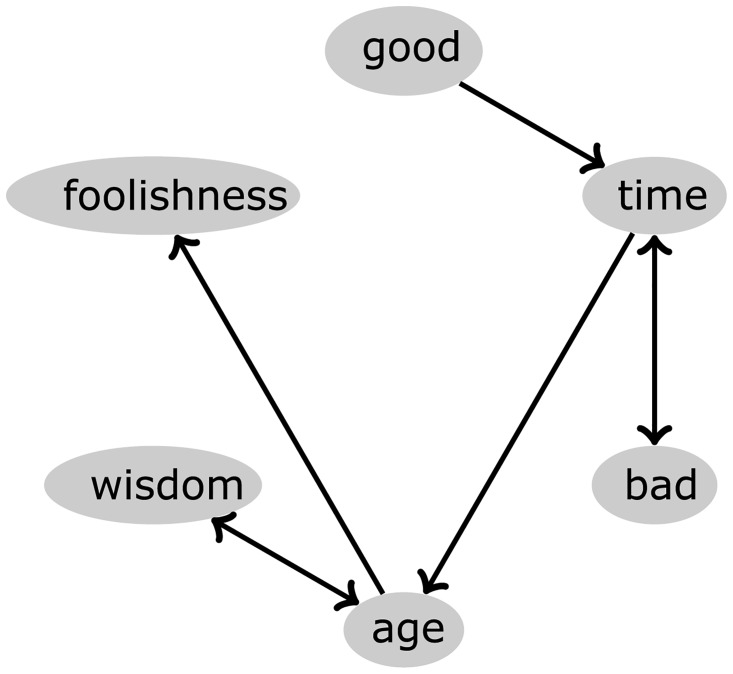
Example of co-occurrence network. The network was obtained for the text “*It was the best of times; it was the worst of times; it was the age of wisdom; it was the age of foolishness*”, which is an extract from the book “A Tale of Two Cities”, by Charles Dickens. Note that, after the removal of stopwords (such as “it” and “was”) and lemmatization process (“times” is mapped to “time”), the remaining words are linked if they are adjacent.

The approach requires a pre-processing step before transforming texts into networks, which consists of the removal of stopwords, and lemmatization: because we are mostly interested in the relationship between content words, stopwords such as function words conveying low semantic information were removed as in many studies of this type [54]. The remaining words were lemmatized so that nouns and verbs were mapped to their singular and infinitive forms, and therefore words related to the same concept were mapped into the same node (also referred to as one single token). Since lemmatization requires part-of-speech (POS) tagging, we used the maximum-entropy approach described in [55]. The co-occurrence networks were constructed with each distinct word becoming a node and two words being linked if they were adjacent in the pre-processed text [34]. The link is directed from the word appearing first to the second word and is weighted by the number of times the pair is found in the text. To illustrate the process of creating a word adjacency network, we show in Fig 1 a network obtained from a short text.

### Characterization of written texts via network dynamics

The proposed method for authorship attribution is based on the evolution of the topology of networks, i.e. we exploit the network dynamics [56]. Therefore, unlike previous approaches (see e.g. [8, 57]), we do not construct one single network from the whole book. Instead, a book is divided into shorter pieces of text comprising the same number of tokens. Then, a co-occurrence network is constructed for each part, which generates a series of independent networks for each book. The last partition is removed from the series because it is shorter than the previous ones. Since distinct books have different numbers of tokens, the series length varies from book to book. This construction guarantees that the remaining networks have the same weight on their edges while the amount of text removed from each book is negligible. Note that we use the same number of tokens in each partition because some network measurements are sensitive to network size. As a consequence, extract comprising different amount of tokens could generate a noise, which would hamper the performance of the classifiers. Note that, in many similar studies where comparing network topology is need, a similar procedure is followed.

Each partition is described by the following topological network measurements: clustering coefficient *C*_*i*_, which gives the fraction of possible triangles that exist for a particular node; network diameter *D*, which is the largest of all longest paths (max{*D*_*ij*_}); network radius *R*, which is the smallest of all longest paths (min{*D*_*ij*_}); number of cliques (complete subgraphs) *C*_*q*_; load centrality *L*_*i*_, similar to betweenness centrality but considering weights on edges; network transitivity *T*, which measures the fraction of all connected triples which are in fact triangles, *T* = 3 × triangles/triads; betweenness centrality *B*_*i*_, which measures how many shortest paths pass through a given node; shortest path length *S*_*ij*_, which is the smallest number of edges between two nodes; degree *K*_*i*_ or connectivity (number of edges) of a node; intermittency *I*_*i*_, which measures how periodically a word is repeated [58]; total number of nodes *N* (i.e. vocabulary size); and total number of edges *E*. Even though intermittency is not a traditional network measurement, we considered it because of its strong relationship with the concept of cycle length in networks. Moreover, this measurement has been proven useful for analyzing text styles [34]. The metrics *D*, *R*, *C*_*q*_, *T*, *N* and *E* are scalar values for a network, while the other measurements are computed for each node individually. In order to have an overall picture of each partition, we computed the average values of *C*_*i*_, *L*_*i*_, *B*_*i*_, *S*_*ij*_, *K*_*i*_ and *I*_*i*_. As such, each partition is characterized by a set of twelve global topological measurements.

The total number of tokens *W* (equal to the total weight on links), in each partition, was selected in a simple optimization procedure, with a compromise between having a long but noisy series (many small networks) and a shorter, more stable one (few large networks). We found that with *W* = 200 tokens one ensures a series length with *T*_*s*_ = 268 elements on average while keeping the number of nodes over *N* = 100 for all networks.

**Fig 2:**
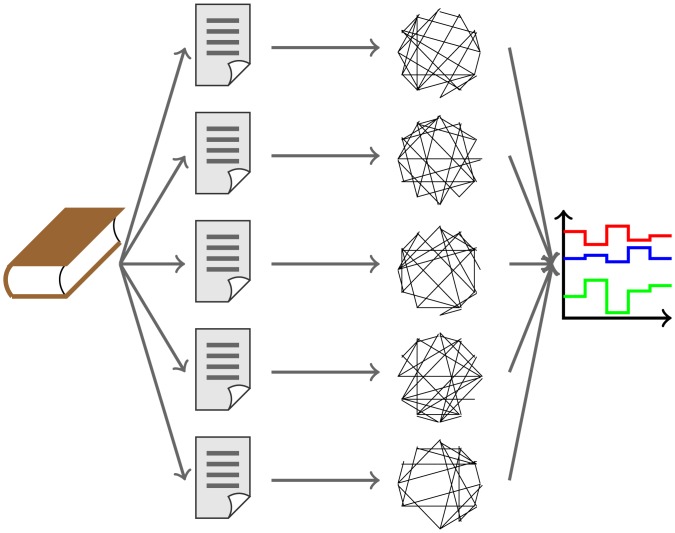
Methodology used to characterize a documents as a set of time series. In the first step, the document is splitted into shorter pieces of equal length. For each subtext, a network is formed. Then, the sequence of networks yield a sequence of complex network measurements. The features extracted from the times series are finally used to identify authorship.

**Fig 3:**
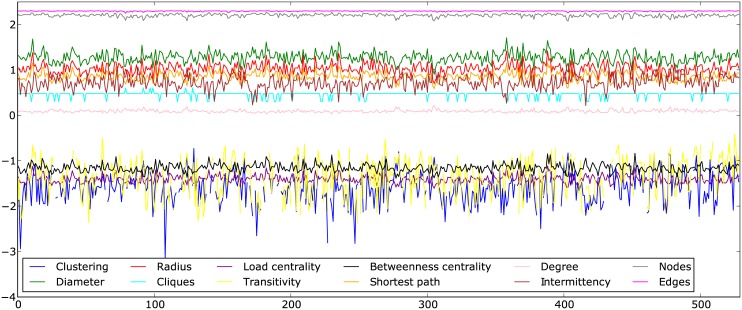
Time series for Moby Dick by Herman Melville. The horizontal axis denotes the index of realizations, and the vertical axis brings the base 10 logarithm of the metrics identified in the inset.

**Fig 4:**
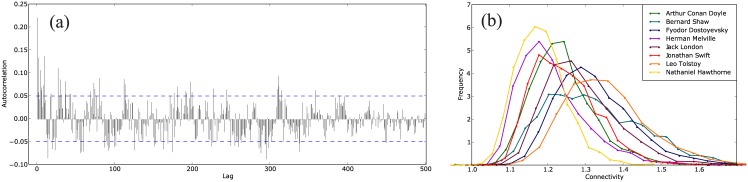
Autocorrelation and histograms for Moby Dick. (a) Autocorrelation for the series of clustering coefficient of Moby Dick by Herman Melville. Dashed lines mark the 5% threshold which is surpassed only by chance. (b) Histograms for time series of degree *K* (connectivity) from all books on the collection grouped by author. The distributions have characteristic moments for each author.

Time series were constructed by extracting the twelve global metrics defined above for each of the networks from a book, as illustrated in Fig 2. Fig 3 shows the series for Moby Dick by Herman Melville, from which one may note that they oscillate steadily around fixed values along the text. Indeed, the analysis is facilitated if the series are stationary. Strong stationarity requires the expected values to be constant throughout while weak stationarity implies that the mean (and possibly the variance) is constant. We confirmed that the time series are stationary, i.e. characterized by low values of autocorrelation. Correlation of a time series with itself shifted by a certain gap measures how much a value in the series depends on the previous ones, implying that autocorrelation must be almost null for all but the first few values of the gap as shown in Fig 4(a). In order to assess series stationarity, we implemented Kwiatkowski-Phillips-Schmidt-Shin (KPSS), augmented Dickey-Fuller, Phillips-Perron, and MacKinnon (finite-sample and asymptotic) tests [59–62]. The null hypothesis of KPSS test is trend stationarity, which for our series was not rejected with 95% confidence (*p*_*value*_ > 0.05) for all metrics. The other tests evaluate the presence of a unit root in the series characteristic equation which is closely related to stationarity; results for these tests are presented in the Supporting Information (S1 File). A standard description of a time series is the fitting into the ARIMA(p, d, q) model. An ARIMA process is described by the relation

$$\left(1-\sum_{i=1}^{p}a_{i}L^{i}\right){\left(1-L\right)}^{d}x_{t}=\left(1+\sum_{i=1}^{q}b_{i}L^{i}\right)\varepsilon_{t}$$ (1)

where *Lx*_*t*_ = *x*_*t*−1_ is the lag operator, implying that if the series is derived *d* times, it becomes stationary and the realization *x*_*t*_ of the series depends on the *p* previous realizations and on the *q* previous independent random variables *ε* (with *t* > *p* + *d* and *t* > *q*). Remarkably, out of the 12 × 80 = 960 time series extracted from the collection, 698 were stationary (*d* = 0) while the remaining series were integrated of first order (*d* = 1), showing that stationarity is a common feature among these series and their derivatives. As for the lag parameters, the maximum order was found for one load centrality series fitted as an ARIMA(5, 0, 4) process (see S1 File).

The finding that the series can be considered stationary allows one to compare estimated values for sample statistics from series of different lengths. The distributions of the series were found to display bell-shaped skewed forms (shown in Fig 4(b)); therefore we propose the first four moments of the series distributions as the dynamical measurements, i.e.

$$\mu_{i}={\left[\frac{1}{T_{s}-1}\sum_{j=1}^{T_{s}}{\left(x_{j}-\mu_{1}\right)}^{i}\right]}^{1/i},$$ (2)

where 1 < *i* ≤ 4 and *μ*_1_ is the average of the measurements in the series. Since there are twelve time series, we obtain 48 dynamical measurements to characterize a book. Note that the distributions in Fig 4(b) are the probability densities of the network global measures which does not contradict the central limit theorem, which states that the sum of independent identically distributed random variables tends to a normal distribution. In other words, with this method the underlying distributions can be determined which would otherwise be lost if the book structure were considered as a whole.

### Data analysis

The moments of the network metrics are used to characterize a book. In the terminology of machine learning these are the attributes (also called features) for the algorithms, while individual books are the instances and the author of a book corresponds to the instance class. A 80 × 48 data matrix is constructed where each row corresponds to an instance and each column corresponds to an attribute. In order to account for the different scales of the attributes (see Fig 3), each column in the data matrix is normalized between zero and one. From the data matrix, the author of each book is inferred using standard supervised learning (classification) algorithms [63].

There is a dimensionality reduction stage prior to the classification. Dimensionality reduction is achieved through either feature selection or feature extraction. Feature selection consists of removing attributes which do not satisfy a given condition, thus leaving a subset or combination of the total number of features. On the other hand, feature extraction blends the original attributes together in order to create a set of, usually fewer, new attributes. Feature selection was implemented using variance threshold and scoring criteria. Variance threshold selection imposes a minimum variance among the realizations of an attribute, for example, if an attribute has the same value for all instances its variance is zero and can be safely removed because it does not contribute to the classification process. We also implemented feature selection based on score. The huge number of combinations of attributes prohibits an exhaustive search of the combination(s) with the highest score. Instead, we start by testing all subsets obtained by removing one attribute from the whole set. In the next step we test all subsets obtained by removing one attribute from the subsets with the highest score in the previous step, and the process is iterated. Dimensionality reduction through feature extraction was implemented using the well-known Principal Component Analysis (PCA) and Isomap [64, 65] techniques. Isomap analyzes data points in a high-dimensional space that are implicitly located in a curved manifold of smaller dimensionality. Dimensionality reduction is then achieved by unwrapping the manifold and getting rid of the extra dimensions. As will be shown, both feature selection and extraction improve the classification success score.

Since there are many supervised learning algorithms, we have selected some to cover the most distinct classification paradigms: ZeroR (0R), which arbitrarily labels all instances as belonging to the most prevalent class; OneR (1R), which ranks attributes according to their error rate and only uses the highest ranked attribute; J48, which organizes the patterns in a tree-like structure; K-nearest neighbors (KNN), where the class of an instance is inferred by a voting process over the nearest neighbors in the training dataset; Naive Bayes (NB), which assumes independence among attributes; and Radial Basis Function Network (RBFN) where a learning network with an input, a processing, and an output layer is used. Due to their simplicity, 0R and 1R are only used for comparison. In all methods, the parameters were set to their default configuration (for KNN *K* = 2 and for RBFN *n*_*clusters*_ = 8) [66] and the classification is calculated for a 10–fold stratified cross-validation [67–70]. A detailed description of classification algorithms can be found in [63].

The performance of algorithms can be evaluated with two standard scores: precision and recall. Both are real values ranging from zero to one, being specific for a given class *c*. Precision (*P*_*c*_) is defined as

$$P_{c}=\frac{\tau_{c}}{\tau_{c}+\epsilon_{c}},$$ (3)

where *τ*_*c*_ is the number of instances belonging to class *c* that were correctly classified (i.e. the number of true positives), and *ϵ*_*c*_ is the number of instances of other classes that were wrongly classified as belonging to class *c* (i.e. number of false positives). The Recall *R*_*c*_ for class *c* is computed as

$$R_{c}=\frac{\tau_{c}}{\tau_{c}+\gamma_{c}},$$ (4)

where *γ*_*c*_ is the number of instances of class *c* that were incorrectly classified (i.e. the number of false negatives). The precision and recall scores defined above refer to a single class. To obtain a single value from the dataset, one may use micro- and macro-averaging. Micro-averaging weights the scores of each class by the number of instances while the macro-average score is the arithmetic mean of the scores of all classes. Note that the micro-averaged recall is equivalent to the success rate, that is, the quotient between the number of instances correctly classified and the total number of instances. For the present collection, having the same number of instances per class, micro- and macro-averaging are equivalent.

## 3 Results and Discussion

**Fig 5:**
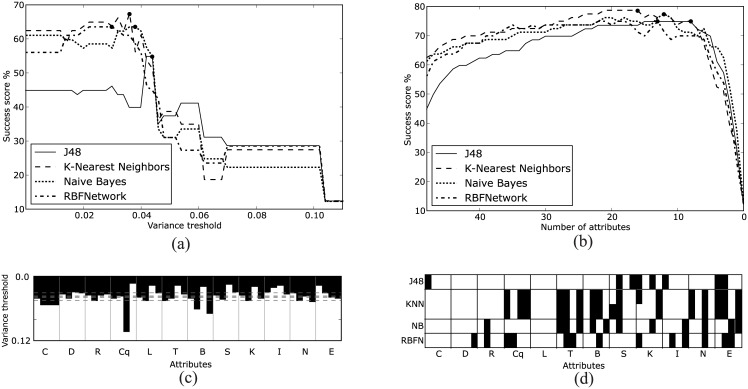
Success scores and combinations of attributes using the variance threshold and score-based feature selection. In (a), (b) the maximum values with minimum number of attributes are marked with circles. In (c), (d) for each network metric (represented by a label in the horizontal axis) the four first moments are presented in increasing order from left to right. A black cell indicates that the attribute is present in the combination. For the variance threshold feature selection, there is a unique combination for every threshold denoted by the vertical axis in (c). For instance, thresholds for the four maximum scores in (a) are marked in (c) by the four dashed horizontal lines. For the score-based feature selection there can be multiple combinations of attributes with the same number of attributes and the same score. Only the combinations with maximum scores and marked with circles in (b) are presented in (d); for KNN algorithm there were two combinations with maximum score.

The authorship signature is captured by the measures proposed, which reveals the relationship between style and changes in network structure. Success scores greatly surpass the threshold in a blind classification obtained with ZeroR algorithm, which for our collection is 1/8 = 12.5%. Unmodified data from the 48 original measures were classified with success rates in the range from 45% to 62.5% as shown in Table 1. The simple OneR algorithm had lower performance, with 35% score. Dimensionality reduction using either feature extraction or feature selection increased the success rates for all algorithms. The results of feature selection are shown in Fig 5 for both variance threshold and score-based selection. In both cases the best results are obtained with an intermediate number of metrics: we begin by removing misleading attributes, therefore improving classification; however, at the end of the process most of the attributes that carry important information are removed and the classification scores drop. In Fig 5(a) and 5(b) success scores are presented, with the maximum value for each curve marked with a circle. If there is more than one maximum (e.g. J48 and NB for variance threshold and J48 and KNN for score-based selection), we only consider the combinations with the fewest number of attributes, located at the rightmost positions.

**Table 1:** Summary of success scores for the original set of 48 attributes and data after feature selection and feature extraction.

Attributes	J48 (%)	KNN (%)	NB (%)	RBFN (%)
Original set	45.00	62.50	61.25	56.25
Variance threshold best	55.00	67.50	63.75	63.75
Score-based best	75.00	78.75	77.50	75.00
{*μ*_1_}	45.00	43.75	46.25	40.00
{*μ*_2_, *μ*_3_, *μ*_4_}	38.75	63.75	60.00	57.50
PCA	40.00	46.25	48.75	42.50
Isomap	63.75	**88.75**	81.25	83.75

The highest scores for feature selection under variance threshold and score-based criteria are presented along with those for the subset of the first moments {*μ*_1_} and the complementary subset of all higher moments {*μ*_1_, *μ*_2_, *μ*_3_}. For PCA and Isomap *n*_*comps*_ = 10 and for Isomap *n*_*neighbors*_ = 10.

The results of feature selection using a variance threshold are shown in Fig 5(a) and 5(c). There is a single subset (combination) of attributes for each variance threshold level. At the lowest threshold in Fig 5(c) all attributes are present and all cells of the highest row are colored black. As the threshold is gradually increased, attributes are successively removed until there are no attributes left and all the cells in the lowest row are colored white. Remarkably, the first and the last attributes removed were respectively the fourth and the third moments of the number of cliques *C*_*q*_. Note also that for nine of the twelve network metrics, either the third or the fourth moment had the smallest variance. The maximum scores are marked with circles in Fig 5(a) and listed in Table 1. The thresholds for maximum scores marked in Fig 5(a) are located in a narrow range and are represented in Fig 5(c) as dashed lines.

The results of feature selection based on score are shown in Fig 5(b) and 5(d). We start with all the attributes in the left end of Fig 5(b). As we explore the combinations obtained by removing one attribute at a time the scores increase (monotonically for J48 and KNN) until a maximum value is reached, after which the scores rapidly decrease reaching ZeroR score when there are zero attributes. The maximum scores are marked with circles in Fig 5(b) and listed in Table 1. It must be noted that the maximum scores can be reached with a few attributes, at most 16 in the case of KNN. The combinations of attributes giving the maximum scores marked are presented in detail in Fig 5(d). For KNN two combinations of attributes reached the highest score. Again, the four moments of a given network metric are grouped together. The best scoring combinations for some algorithms did not include any of the four moments from some network metrics. In particular, load centrality *L* was not used by any algorithm (having therefore a blank column for *L* in Fig 5(d)). One should highlight the betweenness centrality *B*, which was extensively used by KNN, NB and RBFN even though its mean value (i.e. first moment, and the leftmost column under the *B* label on Fig 5(d)) was not used by these algorithms.

Two last combinations of attributes were considered (see 5th and 6th rows of Table 1). The first moments *μ*_1_ represent the static metrics previously studied (see e.g. [34, 71]) and define a subset of 12 attributes. The complementary subset of 36 second, third, and fourth moments represent the dynamical aspects of networks since they describe the extent of variation around the mean value throughout a text. Classification was applied to these two subsets without further dimensionality reduction. The results are listed in the fourth and fifth rows of Table 1 showing that purely dynamical metrics provide better overall performance when compared to the static counterparts, while both subsets score similarly to the whole set of 48 attributes.

**Fig 6:**
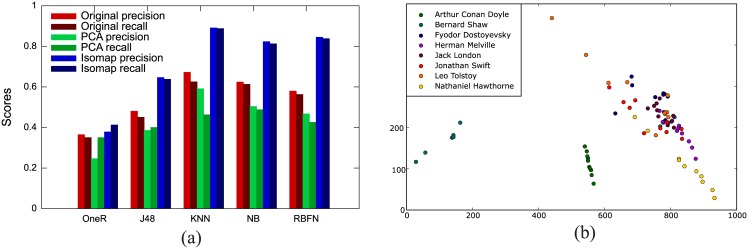
Validation and visualization of complex network measurements. (a) Validation of the classification without dimensionality reduction (red), and with feature extraction using PCA (green) and Isomap (blue). (b) Reduction to two-dimensional attribute space using Isomap. Each point represents a book and each color represents an author.

Another dimensionality reduction technique implemented was feature extraction, using both linear PCA and nonlinear Isomap. The latter uses geodesic distances in an embedded manifold instead of high-dimensional Euclidean distances. There is a free parameter in Isomap: the number of neighbors *n*_*neighbors*_. The distance between two instances considered neighbors is the traditional Euclidean distance while the distance between two other instances is the geodesic distance for a path inside the manifold [64]. The results for Isomap depend on *n*_*neighbors*_ and on the reduced number of dimensions *n*_*comps*_; we varied both parameters from 2 to 15 and found similar results for most cases (see S1 File). The best scores reported below (see Fig 6) were obtained for *n*_*neighbors*_ = 10 and *n*_*comps*_ = 10.

Fig 6(a) shows precision (defined by Eq 3) and recall (success score, defined by Eq 4) for original (without dimensionality reduction), PCA-, and Isomap-treated attributes (see also Table 1). Dimensionality reduction through PCA leads to lower precision and recall, while Isomap enhances the classification efficacy of the algorithms. The best performance is reached with KNN for which the authorship of 71 out of the 80 texts in the collection is correctly identified, thus reaching 88.75% success score (recall) and 0.891 precision. To our knowledge, this is the best performance obtained with a technique based solely on topological properties of complex networks. This is also the first time that Isomap has shown to be useful to improve the performance of the classification of this type of data. In fact, while the original Isomap may not be adequate for classification tasks owing to the use of the reconstruction principle [72], extended versions of this visualization technique may provide a better discrimination of the original data [72, 73]. This performance is robust among algorithms since both precision and recall surpass 80% using KNN, NB and RBFN. For visualization purposes Isomap was also applied to reduce the number of attributes to a two-dimensional space using the Projection Explorer software [65] as shown in Fig 6(b). For some authors the texts are clearly grouped and separated from the rest (e.g. texts from A. C. Doyle and B. Shaw) while for other authors the separation is not as clear. A common trend exists nevertheless, with texts by the same author located in preferential regions in the attribute space. However, a much better separation could be obtained if higher dimensions were considered, as confirmed by the high accuracy rate obtained with the Isomap projection.

Even though a direct comparison to related works requires using the same texts collection, two examples using collections with similar characteristics which use static network metrics are worth mentioning. A similar study for the same task [34] analyzed 40 texts from 8 authors in English reaching a success score of 65%. In another work, 28 out of 36 Persian books from 5 authors were correctly classified [71]. A myriad of other features for authorship identification have been proposed. Argamon and Juola [74] collected the results of the PAN 2011 competition where 3001 electronic messages from 26 authors were classified using diverse features for which the best micro-averaged recall (i.e. success score) was 0.717. These collections have characteristics different to ours such as the number of texts, authors, and the sizes of messages compared to books. To our knowledge, ours is the best performance achieved to date using only topological features of co-occurrence networks.

## 4 Conclusion

Network sample statistics could be used for classifying texts in a straightforward manner owing to the stationarity of the series obtained with the network metrics; as a bonus, problems faced in applying networks to real-life situations were solved. For instance, texts of different sizes could be compared, and indeed, the smallest book of the collection (from A. C. Doyle) was correctly classified repeatedly regardless of the small size of its series. It is possible to compare texts of dissimilar sizes because the size of a book is reflected on the length of the corresponding time series rather than on the sizes of the networks. Also, the finite-size effects of typical networks from language are avoided by considering only the mean values over a whole network. The typical sizes of the networks were slightly more than 100 nodes, which are usually considered small; however, our approach succeeds because it collects only global metrics, i.e. averages that are still reliable, in contrast to distributions over all nodes. This trading of the microscopic description of each node of a network for the sample statistics of the global metrics resulted in a fair description of the latter as shown in Fig 4(b) owing to the length of the series. It remains to be shown to which extent the microscopic distributions over the nodes depend on the global averages and on the growth mechanisms as it has been claimed using theoretical models [29, 30].

Even though the primary goal of the work was not to compete with other approaches in authorship recognition, but rather to contribute to understanding the influence of authorship on the statistical properties of co-occurrence networks, the method proposed has proven reliable for authorship identification. Success scores reached 88.75% (KNN+Isomap), which is outstanding for a collection with this particular number of books and authors. Indeed, our method is among the most successful authorship recognition approaches according to the literature [74]. For instance, the attributes proposed are more difficult to be attacked because they depend on text structure instead of text formatting or simple frequency statistics [75, 76] making them hard to forge. Also, network methods are in general less sensitive to topic and are therefore more suitable to study writing styles [33, 34, 71]. Computational complexity is much lower than that of traditional frequency-based methods as the method is less demanding for the learning algorithms; it is also less computationally expensive than previous network-based methods because the complexity of calculation of most network metrics scales faster than linearly with the number of nodes. The present method is flexible to include other network features for optimizing classification and to find hidden relations among measures. The robustness of results with various classification algorithms supports the reliability of the measures proposed and opens the prospect of finding optimal algorithms and parameter values that further improves classification.

With regard to the failures in classification, we noted that small books in the collection are not the source of wrong classification. We conclude that the mistakes are caused by the variability of style of authors among their books: while for some authors texts are clearly concentrated in a small region of attribute space, the texts from others are scattered. This reflects that some authors use well-defined structures while others change their narrative resources from one text to another. Dimensionality reduction through non-linear feature extraction helped to raise the success rates in classification while linear feature extraction scored worse reflecting the non-trivial relations among the network metrics and the irregular distribution of instances in the attribute space.

Converting networks structural information to series of measures allows one to apply time series analysis to the study of the evolution of network topologies, and in particular, to the way an author uses the structures offered by the language in his/her narrative. Purely dynamical measures, i.e. higher moments of the time series, revealed an aspect hitherto unknown of the close relation between style and network dynamics. An author controls not only the macroscopic structure of the whole text but also the extent to which different metrics can be modified independently, reflecting the richness of language use in terms of co-occurrence networks.

## Supporting Information

S1 File List of books employed in the experiments. (PDF)
