# Supplementary material for: Text Authorship Identified Using the Dynamics of Word Co-Occurrence Networks
Source: PLoS One. 2017 Jan 26;12(1):e0170527. doi: 10.1371/journal.pone.0170527 (PMC5268788; doi:10.1371/journal.pone.0170527)
Supplement: S1 File — (PDF) [file pone.0170527.s001.pdf]

Supporting Information

For the article: Text authorship identified using the dynamics of word co-occurrence networks

Collection of texts

**Arthur Conan Doyle**	
A Study in Scarlet	gutenberg.org/ebooks/244
His Last Bow: An Epilogue of Sherlock Holmes	gutenberg.org/ebooks/2350
The Adventures of Sherlock Holmes	gutenberg.org/ebooks/1661
The Gully of Bluemansdyke	gutenberg.org/ebooks/40848
The Hound of the Baskervilles	gutenberg.org/ebooks/2852
The Lost World	gutenberg.org/ebooks/139
The Memoirs of Sherlock Holmes	gutenberg.org/ebooks/834
The Return of Sherlock Holmes	gutenberg.org/ebooks/221
The Sign of the Four	gutenberg.org/ebooks/2097
The Valley of Fear	gutenberg.org/ebooks/3289
**Bernard Shaw**	
Major Barbara	gutenberg.org/ebooks/3790
Man and Superman: A Comedy and a Philosophy	gutenberg.org/ebooks/3328
Mrs. Warren's Profession	gutenberg.org/ebooks/1097
Pygmalion	gutenberg.org/ebooks/3825
Caesar and Cleopatra	gutenberg.org/ebooks/3329
Cashel Byron's Profession	gutenberg.org/ebooks/5872
John Bull's Other Island	gutenberg.org/ebooks/3612
An Unsocial Socialist	gutenberg.org/ebooks/1654
On the Prospects of Christianity	gutenberg.org/ebooks/4004
Treatise on Parents and Children	gutenberg.org/ebooks/908
**Fyodor Dostoyevsky**	
Short Stories	gutenberg.org/ebooks/40745
Crime and Punishment	gutenberg.org/ebooks/2554
Notes from the Underground	gutenberg.org/ebooks/600
Poor Folk	gutenberg.org/ebooks/2302
The Brothers Karamazov	gutenberg.org/ebooks/28054
The Gambler	gutenberg.org/ebooks/2197
The Idiot	gutenberg.org/ebooks/2638
The Possessed (The Devils)	gutenberg.org/ebooks/8117
Uncle's Dream; and The Permanent Husband	gutenberg.org/ebooks/38241
White Nights and Other Stories	gutenberg.org/ebooks/36034
**Herman Melville**	
Mardi: and A Voyage Thither, Vol. I	gutenberg.org/ebooks/13720
Mardi: and A Voyage Thither, Vol. II	gutenberg.org/ebooks/13721
Moby Dick; Or, The Whale	gutenberg.org/ebooks/2701
Omoo: Adventures in the South Seas	gutenberg.org/ebooks/4045
Pierre; or The Ambiguities	gutenberg.org/ebooks/34970
Redburn. His First Voyage	gutenberg.org/ebooks/8118
The Confidence-Man: His Masquerade	gutenberg.org/ebooks/21816
The Piazza Tales	gutenberg.org/ebooks/15859
Typee: A Romance of the South Seas	gutenberg.org/ebooks/1900
White Jacket; Or, The World on a Man-of-War	gutenberg.org/ebooks/10712

**Jack London**	
Burning Daylight	gutenberg.org/ebooks/746
Martin Eden	gutenberg.org/ebooks/1056
Michael, Brother of Jerry	gutenberg.org/ebooks/1730
Smoke Bellew	gutenberg.org/ebooks/1596
The Iron Heel	gutenberg.org/ebooks/1164
The Jacket (The Star-Rover)	gutenberg.org/ebooks/1162
The Little Lady of the Big House	gutenberg.org/ebooks/6455
The Mutiny of the Elsinore	gutenberg.org/ebooks/2415
The Sea-Wolf	gutenberg.org/ebooks/1074
The Valley of the Moon	gutenberg.org/ebooks/1449
**Jonathan Swift**	
Gulliver's Travels into Several Remote Nations of the World	gutenberg.org/ebooks/829
The Journal to Stella	gutenberg.org/ebooks/4208
The Poems of Jonathan Swift, D.D., Volume 1	gutenberg.org/ebooks/14353
The Poems of Jonathan Swift, D.D., Volume 2	gutenberg.org/ebooks/13621
The Prose Works of Jonathan Swift, D.D. Volume 03	gutenberg.org/ebooks/12252
The Prose Works of Jonathan Swift, D.D. Volume 04	gutenberg.org/ebooks/12746
The Prose Works of Jonathan Swift, D.D. Volume 06	gutenberg.org/ebooks/12784
The Prose Works of Jonathan Swift, D.D. Volume 07	gutenberg.org/ebooks/18250
The Prose Works of Jonathan Swift, D.D. Volume 09	gutenberg.org/ebooks/13169
The Prose Works of Jonathan Swift, D.D. Volume 10	gutenberg.org/ebooks/13040
**Leo Tolstoy**	
Anna Karenina	gutenberg.org/ebooks/1399
Redemption and two other plays	gutenberg.org/ebooks/9792
Resurrection	gutenberg.org/ebooks/1938
The Awakening	gutenberg.org/ebooks/17352
The Cossacks: A Tale of 1852	gutenberg.org/ebooks/4761
The Kingdom of God Is Within You	gutenberg.org/ebooks/4602
War and Peace	gutenberg.org/ebooks/2600
What Shall We Do?	gutenberg.org/ebooks/38690
What to Do? Thoughts Evoked by the Census of Moscow	gutenberg.org/ebooks/3630
Youth	gutenberg.org/ebooks/2637
**Nathaniel Hawthorne**	
True Stories of History and Biography	gutenberg.org/ebooks/15697
The Scarlet Letter	gutenberg.org/ebooks/33
The Marble Faun; Or, The Romance of Monte Beni - Volume 2	gutenberg.org/ebooks/2182
Doctor Grimshawe's Secret	gutenberg.org/ebooks/7183
Passages from the English Notebooks, Complete	gutenberg.org/ebooks/7878
Passages from the French and Italian Notebooks, Volume 1	gutenberg.org/ebooks/7879
Passages from the French and Italian Notebooks, Volume 2	gutenberg.org/ebooks/7880
Our Old Home: A Series of English Sketches	gutenberg.org/ebooks/8090
Sketches and Studies	gutenberg.org/ebooks/8091
Twice Told Tales	gutenberg.org/ebooks/13707

Success scores using Isomap

The results of classification after applying Isomap for the various classification algorithms are presented as histograms in figure S1, where each figure includes the 196 cases obtained by varying both $n_{components}$ and $n_{neighbors}$ from 2 to 15. For all algorithms the histograms are leaned to the right, reflecting that in the majority of cases a high score is obtained. The maximum value was 88.75% obtained with KNN as shown in figure S1b. For this classification algorithm the results are presented in detail in table 1.

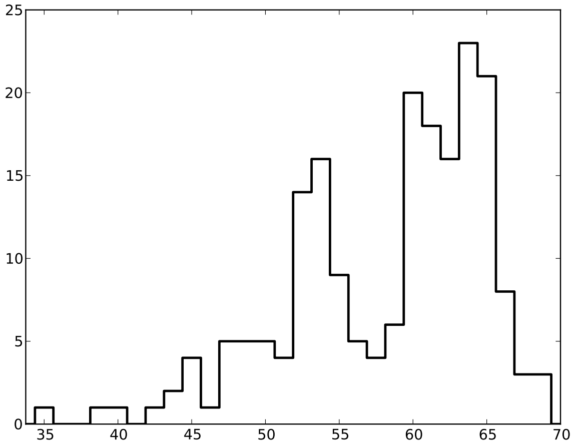

(a) J48 scores (%).

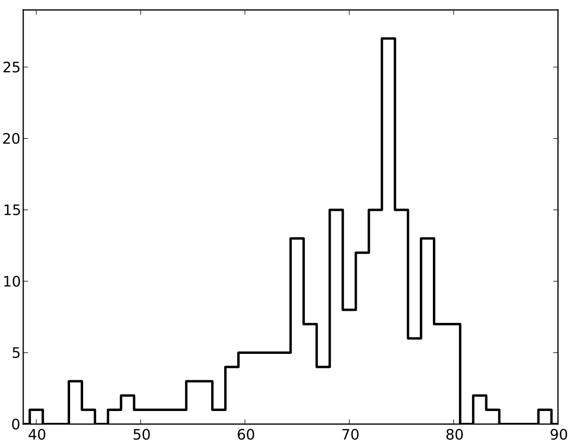

(b) KNN scores (%).

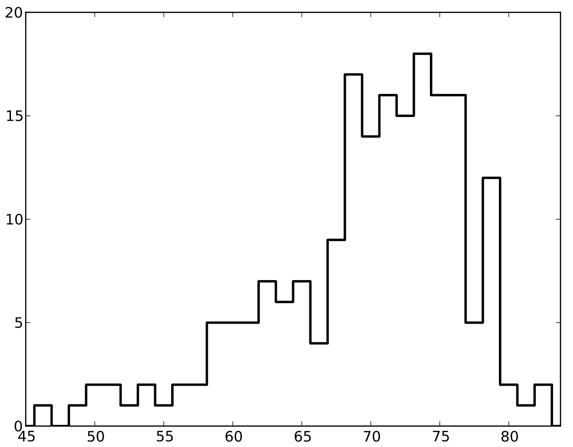

(c) Naive Bayes scores (%).

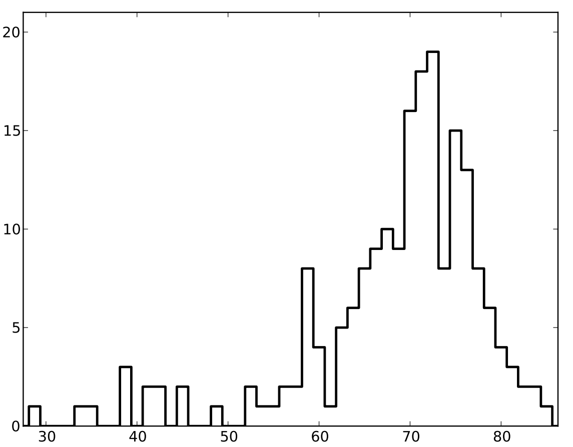

(d) RBFNetwork scores (%).

Figure S 1: Success scores after applying Isomap. Parameters $n_{components}$ and $n_{neighbors}$ of Isomap were varied from 2 to 15 giving a total of 196 scores. Histograms show a similar behavior for most values of the parameters having a peak at the right side of each figure and therefore high values for the scores.

Table S 1: Scores obtained with KNN after applying Isomap for different values of the parameters $n_{components}$ and $n_{neighbors}$.

n_{comp}	n_{neigh}	Score
2	2	53.75
2	3	72.50
2	4	48.75
2	5	52.50
2	6	65.00
2	7	58.75
2	8	47.50
2	9	45.00
2	10	48.75
2	11	43.75
2	12	43.75
2	13	40.00
2	14	50.00
2	15	43.75
3	2	61.25
3	3	71.25
3	4	51.25
3	5	55.00
3	6	67.50
3	7	73.75
3	8	78.75
3	9	70.00
3	10	80.00
3	11	70.00
3	12	71.25
3	13	73.75
3	14	55.00
3	15	56.25
4	2	60.00
4	3	68.75
4	4	62.50
4	5	66.25
4	6	73.75
4	7	76.25
4	8	77.50
4	9	71.25
4	10	76.25
4	11	67.50
4	12	71.25
4	13	72.50
4	14	56.25
4	15	60.00
5	2	65.00
5	3	68.75
5	4	55.00
5	5	65.00
5	6	75.00
5	7	73.75
5	8	76.25
5	9	68.75
5	10	77.50
5	11	71.25
5	12	75.00
5	13	72.50
5	14	63.75
5	15	65.00
6	2	62.50
6	3	73.75
6	4	56.25
6	5	65.00
6	6	71.25
6	7	77.50
6	8	77.50
6	9	72.50
6	10	78.75
6	11	70.00
6	12	75.00
6	13	73.75
6	14	62.50
6	15	65.00
7	2	63.75
7	3	73.75
7	4	58.75
7	5	75.00
7	6	68.75
7	7	78.75
7	8	73.75
7	9	72.50
7	10	77.50
7	11	68.75
7	12	75.00
7	13	73.75
7	14	63.75
7	15	65.00
8	2	68.75
8	3	75.00
8	4	63.75
8	5	76.25
8	6	68.75
8	7	83.75
8	8	73.75
8	9	71.25
8	10	78.75
8	11	75.00
8	12	70.00
8	13	72.50
8	14	61.25
8	15	65.00
9	2	72.50
9	3	76.25
9	4	68.75
9	5	75.00
9	6	73.75
9	7	80.00
9	8	75.00
9	9	72.50
9	10	78.75
9	11	78.75
9	12	72.50
9	13	75.00
9	14	60.00
9	15	68.75
10	2	73.75
10	3	77.50
10	4	67.50
10	5	73.75
10	6	73.75
10	7	80.00
10	8	70.00
10	9	72.50
**10**	**10**	**88.75**
10	11	78.75
10	12	73.75
10	13	73.75
10	14	60.00
10	15	68.75
11	2	65.00
11	3	77.50
11	4	66.25
11	5	77.50
11	6	73.75
11	7	80.00
11	8	72.50
11	9	73.75
11	10	80.00
11	11	73.75
11	12	72.50
11	13	71.25
11	14	58.75
11	15	66.25
12	2	65.00
12	3	75.00
12	4	66.25
12	5	68.75
12	6	75.00
12	7	80.00
12	8	71.25
12	9	71.25
12	10	76.25
12	11	73.75
12	12	68.75
12	13	68.75
12	14	61.25
12	15	65.00
13	2	63.75
13	3	77.50
13	4	71.25
13	5	61.25
13	6	73.75
13	7	77.50
13	8	72.50
13	9	73.75
13	10	82.50
13	11	73.75
13	12	70.00
13	13	66.25
13	14	60.00
13	15	66.25
14	2	62.50
14	3	77.50
14	4	68.75
14	5	61.25
14	6	73.75
14	7	77.50
14	8	73.75
14	9	73.75
14	10	82.50
14	11	72.50
14	12	70.00
14	13	65.00
14	14	57.50
14	15	66.25
15	2	65.00
15	3	80.00
15	4	68.75
15	5	62.50
15	6	72.50
15	7	77.50
15	8	75.00
15	9	75.00
15	10	75.00
15	11	70.00
15	12	73.75
15	13	71.25
15	14	58.75
15	15	67.50

Tests results

The features of a time series can be tested with the so-called unit root tests. These tests assume a null hypothesis and verify whether the hypothesis can be rejected in which case an alternative hypothesis is considered. Each one of the tests has a particular null hypothesis and alternative hypothesis, and each test considers different evidences to accept or reject the hypotheses. The Kwiatkowski-Phillips-Schmidt-Shin (KPSS) test is the only one that considers trend-stationarity as the null hypothesis, the alternative hypothesis being the presence of a unit root. For the Phillips-Perron test, the null hypothesis is that the series is integrated of order 1. We considered also the Augmented Dickey-Fuller test for which the null hypothesis is the presence of a unit root. Finally, McKinnon also considers the presence of a unit root as its null hypothesis but uses alternative distributions to estimate the p_{value} (columns 5 and 6 in table 2 correspond to finite sample and asymptotic tests). It must be noted that the results obtained in table 2 are averages over the 80 books instead of the results for a single series.

Table S 2: Averages of p_{values} obtained with various tests.

	**Phillips-Perron**	**KPSS**	**Dickey-Fuller**	**McKinnon**	
Clustering	0.010	0.071	0.017	0.008	0.167
Betweenness centrality	0.023	0.074	0.350	0.360	0.510
Cliques	0.010	0.086	0.377	0.393	0.521
Diameter	0.010	0.076	0.116	0.111	0.365
Intermittency	0.010	0.071	0.080	0.074	0.335
Load centrality	0.081	0.080	0.457	0.478	0.583
Degree	0.019	0.066	0.470	0.513	0.579
Radius	0.011	0.073	0.118	0.114	0.368
Shortest path	0.013	0.071	0.214	0.208	0.430
Edges	0.253	0.078	0.362	0.369	0.512
Nodes	0.022	0.067	0.368	0.378	0.514
Transitivity	0.010	0.083	0.014	0.005	0.126

ARIMA fittings

The $80 \times 12 = 960$ series were fitted as ARIMA(p,d,q) processes. Each table corresponds to a parameter and each entry in the table corresponds to the number of series that were fitted with the parameter set to the value indexed by the column. Table 6 corresponds to the sum $p + d + q$.

Table S 3: Number of series fitted with an ARIMA(p,d,q) model for each value of parameter p.

Network metric	Value of p									
	0	1	2	3	4	5	6	7	8	9
Clustering	36	27	11	5	1	0	0	0	0	0
Betweenness centrality	25	37	12	6	0	0	0	0	0	0
Cliques	59	11	7	2	1	0	0	0	0	0
Diameter	32	32	8	8	0	0	0	0	0	0
Intermittency	27	34	13	5	1	0	0	0	0	0
Load centrality	35	28	9	7	0	1	0	0	0	0
Degree	28	36	11	3	1	1	0	0	0	0
Radius	27	32	14	7	0	0	0	0	0	0
Shortest path	25	26	21	4	3	1	0	0	0	0
Edges	40	25	8	6	1	0	0	0	0	0
Nodes	27	33	13	5	1	1	0	0	0	0
Transitivity	51	20	6	1	2	0	0	0	0	0
Total	412	341	133	59	11	4	0	0	0	0

Table S 4: Number of series fitted with an ARIMA(p,d,q) model for each value of parameter d.

Network metric	Value of d									
	0	1	2	3	4	5	6	7	8	9
Clustering	55	25	0	0	0	0	0	0	0	0
Betweenness centrality	57	23	0	0	0	0	0	0	0	0
Cliques	69	11	0	0	0	0	0	0	0	0
Diameter	60	20	0	0	0	0	0	0	0	0
Intermittency	56	24	0	0	0	0	0	0	0	0
Load centrality	63	17	0	0	0	0	0	0	0	0
Degree	51	29	0	0	0	0	0	0	0	0
Radius	58	22	0	0	0	0	0	0	0	0
Shortest path	55	25	0	0	0	0	0	0	0	0
Edges	61	19	0	0	0	0	0	0	0	0
Nodes	49	31	0	0	0	0	0	0	0	0
Transitivity	64	16	0	0	0	0	0	0	0	0
Total	698	262	0	0	0	0	0	0	0	0

Table S 5: Number of series fitted with an ARIMA(p,d,q) model for each value of parameter q.

Network metric	Value of q									
	0	1	2	3	4	5	6	7	8	9
Clustering	31	29	13	6	1	0	0	0	0	0
Betweenness centrality	16	42	16	6	0	0	0	0	0	0
Cliques	53	16	10	1	0	0	0	0	0	0
Diameter	27	36	11	5	1	0	0	0	0	0
Intermittency	21	38	19	2	0	0	0	0	0	0
Load centrality	32	33	10	4	1	0	0	0	0	0
Degree	21	44	12	2	0	1	0	0	0	0
Radius	27	32	18	3	0	0	0	0	0	0
Shortest path	23	40	10	6	1	0	0	0	0	0
Edges	35	29	10	4	1	1	0	0	0	0
Nodes	26	40	11	3	0	0	0	0	0	0
Transitivity	39	27	13	1	0	0	0	0	0	0
Total	351	406	153	43	5	2	0	0	0	0

Table S 6: Number of series fitted with an ARIMA(p,d,q) model for each value of the sum $p+d+q$.

Network metric	Value of $p+d+q$									
	0	1	2	3	4	5	6	7	8	9
Clustering	20	15	16	10	12	2	2	1	2	0
Betweenness centrality	9	9	27	19	9	4	3	0	0	0
Cliques	42	14	11	5	6	2	0	0	0	0
Diameter	13	20	20	11	9	4	1	2	0	0
Intermittency	8	20	17	17	11	6	1	0	0	0
Load centrality	18	19	17	14	4	5	1	1	0	1
Degree	8	16	24	16	10	5	0	1	0	0
Radius	13	16	20	12	11	5	2	1	0	0
Shortest path	8	18	20	11	12	3	6	2	0	0
Edges	21	21	12	10	9	4	1	1	1	0
Nodes	9	19	21	13	10	4	3	1	0	0
Transitivity	32	16	13	9	7	1	1	1	0	0
Total	201	203	218	147	110	45	21	11	3	1

Table S 7: Series fitted with an ARIMA(p,d,q) model having the biggest values of the sum $p+d+q$.

			ARIMA(p,d,q)		
Book	Measure	Sum	p	d	q
The Poems of Jonathan Swift, D.D., Volume 2	Load centrality	9	5	0	4
The Journal to Stella	Clustering	8	2	1	5
The Iron Heel	Clustering	8	3	1	4
Typee: A Romance of the South Seas	Edges	8	4	1	3
